# A Peculiar Case of Hydatid Disease

**DOI:** 10.7759/cureus.20729

**Published:** 2021-12-27

**Authors:** Gaurav Singla, Anchal Sharma, Yogita Nevtia

**Affiliations:** 1 Radiodiagnosis, Maharishi Markandeshwar Medical College and Hospital, Solan, IND; 2 Radiodiagnosis, Dayanand Medical College and Hospital, Ludhiana, IND

**Keywords:** hepatic hydatid cyst, pulmonary hydatid cyst, hydatid disease, ct, usg, ncct, mri

## Abstract

Hydatid disease primarily affects the liver which is the most common location. This article highlights a rare representation of the hydatid disease which has led to hematogenous spread of the disease and gives us a wider picture that how a common disease can have an uncommon presentation. Another point that is addressed in this research article is that the widely accepted classification systems for hydatid disease can be modified even further, making them even more accurate. Here, we present a case of a 32-year-old male (non-smoker and non-alcoholic) who presented with focal neurological deficit, diffuse headache, diffuse abdominal pain, and breathing difficulties for the past six months. The patient is a known farmer and lives in an agricultural sheep-grazing area. With the help of MRI brain and non-contrast CT (NCCT) chest and abdomen, it was confirmed to be a case of multiple hepatic hydatid cysts in various stages, with transdiaphragmatic spread to adjacent lung and cerebral hydatidosis as evident by focal neurological deficit. No history of seizures has been given by the patient.

## Introduction

Life cycle in humans

*Echinococcus granulosus* (*E. granulosus*) is a tapeworm of dogs; the tapeworm eggs are passed in the feces of dogs and then ingested by an intermediate host, commonly in sheep during grazing, where they develop and grow into cystic structures [1]. After ingestion of the cysts by carnivorous dogs, the life cycle is completed and numerous tapeworms develop in the intestine of the definitive host. Humans act as accidental intermediate hosts by consuming the contaminated vegetables from the feces of the definitive host containing the eggs; this harbor cysts inside an accidental host, most commonly in the liver via the portal venous and the lymphatic system through the intestinal wall, however, echinococcal cysts may develop in almost any part of the body except hair, teeth, and fingernails [2]. The liver is the most common site of infection, followed by the lung in 15-25% of the cases, and other sites (spleen, kidney, brain, bone) in about 10% of the cases [3-5].

Epidemiology

Hydatid disease is a global zoonotic infection caused by Echinococcus in its larval stage. Hydatid disease is caused by *E. granulosus* and *Echinococcus multilocularis* which are the two main types. *E. granulosus* is mainly seen in New Zealand, Australia, the Middle East, and Africa which makes it the most common type of hydatid disease in humans [1-3]. The classical findings are well known, however, findings related to unusual anatomic locations and extensions are less frequently described in the literature [6]. Here, we present an interesting case of hepatic hydatid cyst with exophytic growth and transdiaphragmatic thoracic involvement with likely impending rupture.

Role of ultrasound and computed tomography (CT)

Ultrasound is the primary modality of diagnosis but CT scan can help in various cases like excessive abdominal gas, high build status, and history of previous surgeries or disease extensions (communication with the biliary tree). CT has high sensitivity and specificity for hepatic hydatid disease [6]. CT scan help in the diagnosis of hydatid cyst by showing the high attenuation wall of hydatid cyst which can be seen both when it is calcified or even without calcification, but certain setbacks like in cases of contrast enhancement, hemochromatosis, and drug therapy (like amiodarone, etc.) can increase hepatic attenuation which can obscure visualization of wall of the hepatic hydatid cyst [6]. So non-contrast CT (NCCT) is better than contrast-enhanced CT scan as contrast can obscure the high attenuation cyst wall. A free-floating membrane within the cyst seen as high attenuation membrane is the peri-cystic laminated membrane of hydatid. Multiple, round peripherally arranged vesicular structures within the main cyst are the daughter cysts that contain cystic fluid of low attenuation as compared to the main cyst [6].

## Case presentation

**Figure 1:**
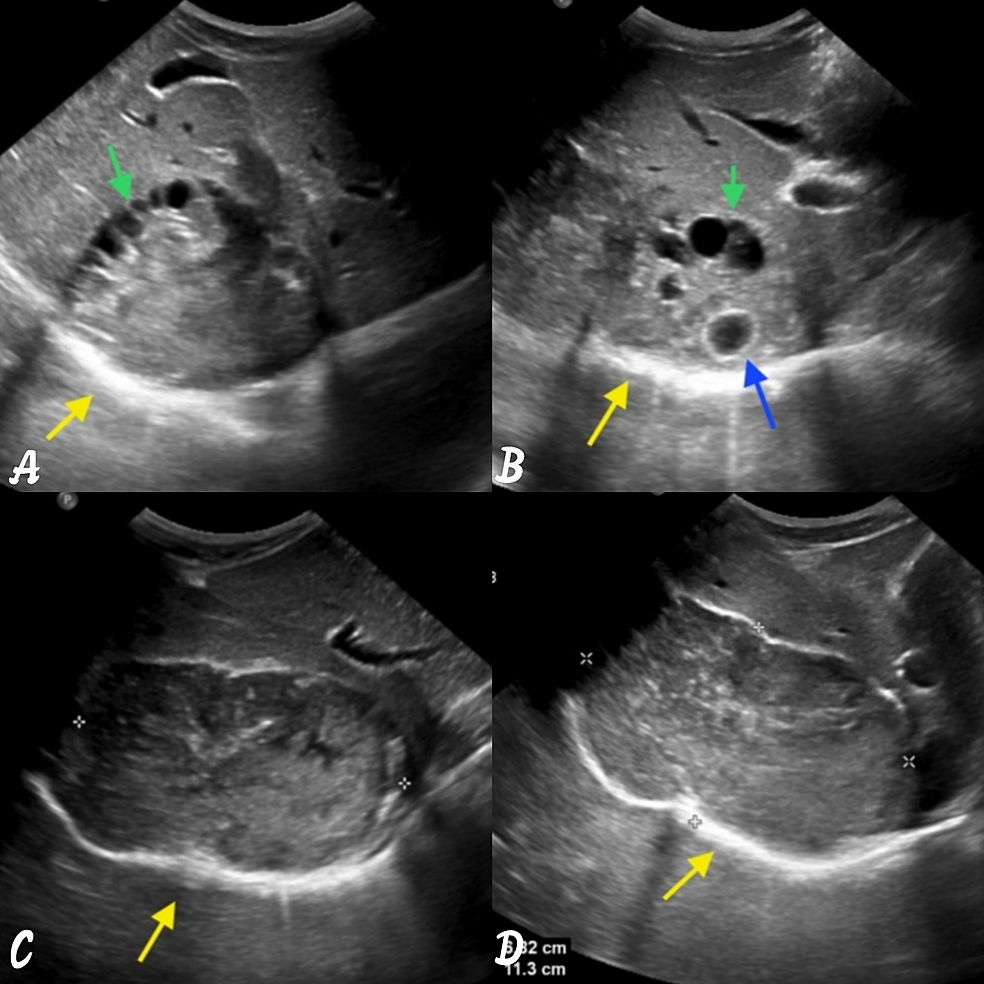
Ultrasonographic images showing multiple daughter cysts. (A) The cysts are located along the periphery giving spoke wheel appearance (green arrow). (B) Few of the daughter cysts show echogenic walls likely due to calcification (blue arrow). (C and D) The main cyst shows heterogeneous echotexture and is abutting the right hemidiaphragm (yellow arrow) and lies in segments VII and VIII.

**Figure 2:**
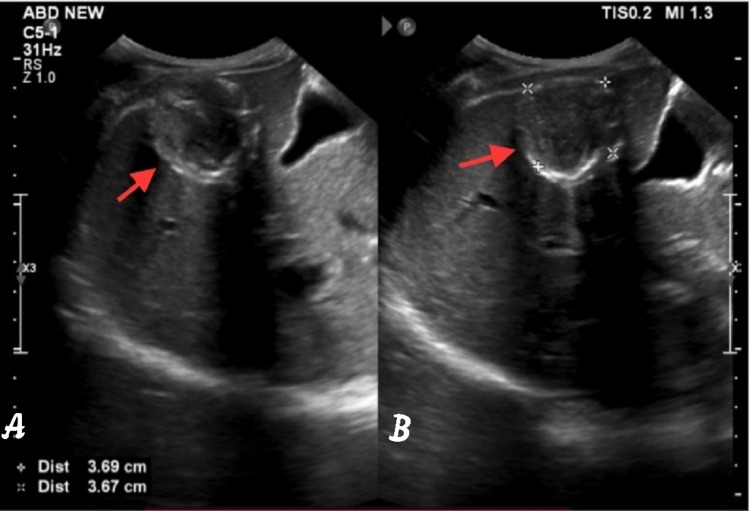
Image is showing stage 5 of Gharbi classification (inactive/dead hydatid cyst). (A) Complete whorl of calcification forming a complete ring and giving posterior acoustic shadowing from the anterior wall (red arrow). (B) The cyst measures 3.7x3.6 cm.

A 32-year-old male, farmer by occupation, presented with focal neurological deficit and headache with diffuse abdominal pain for the past six months associated with difficulty in breathing. The patient has a history of contact with dogs and farm animals and lives in an agricultural sheep-grazing area. The patient is non-alcoholic and non-smoker. Hematological studies revealed leucocytosis and eosinophilia with mildly raised alkaline phosphatase. Serologic echinococcal test is falsely negative. So, to rule out any pathology, ultrasound whole abdomen is advised which shows a large heterogeneous echogenicity cyst with a few hypoechoic cysts in the liver in segments IVA, VII, and VIII (Figures 1A-1D). Ultrasonography shows that the lesion is abutting the right hemidiaphragm (Figures 1A-1D). Another calcified cyst in the segment V of the liver is shown in Figures 2A, 2B.

**Figure 3:**
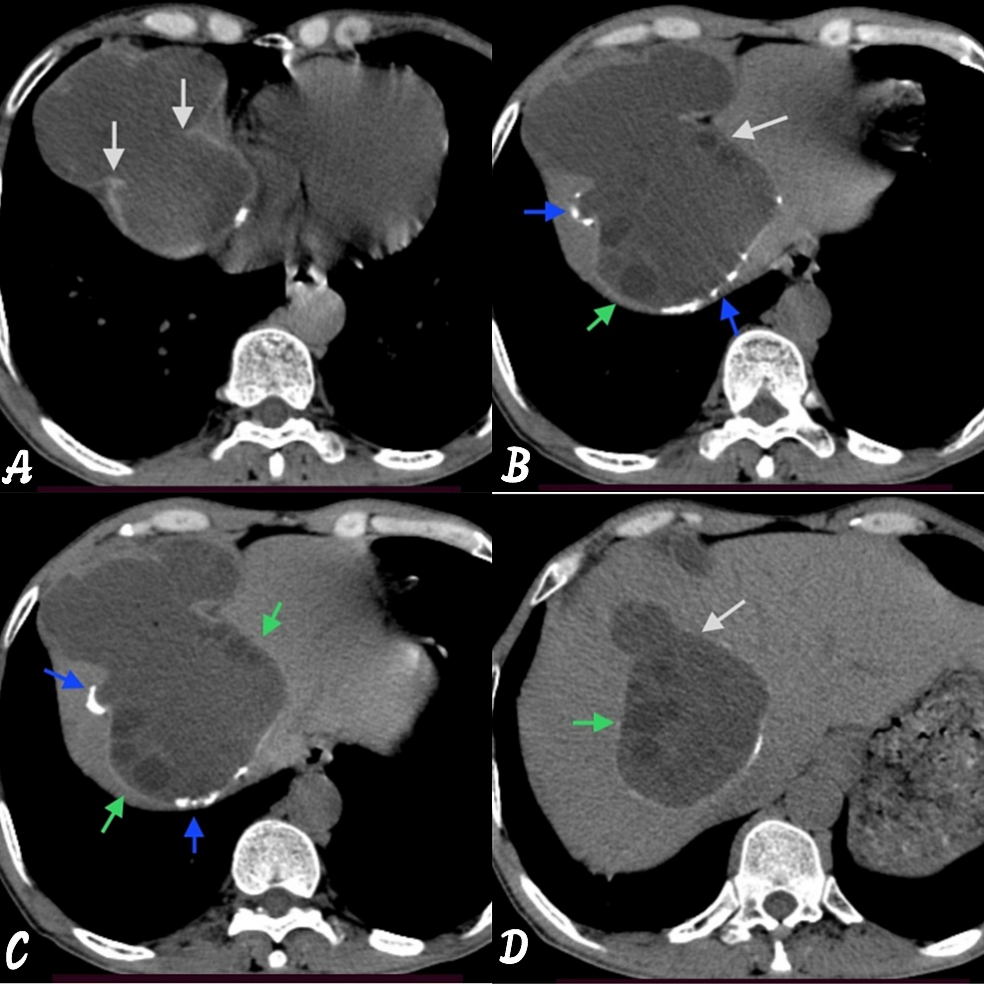
Axial CT images showing hydatid cyst reaching up to right atrium. Axial images showing (A) incomplete membrane within the hydatid cyst (white arrow); (B and C) incomplete calcifications (blue arrow) and daughter cyst (green arrow); and (D) incomplete membrane within the hydatid cyst (white arrow) and daughter cyst (green arrow).

**Figure 4:**
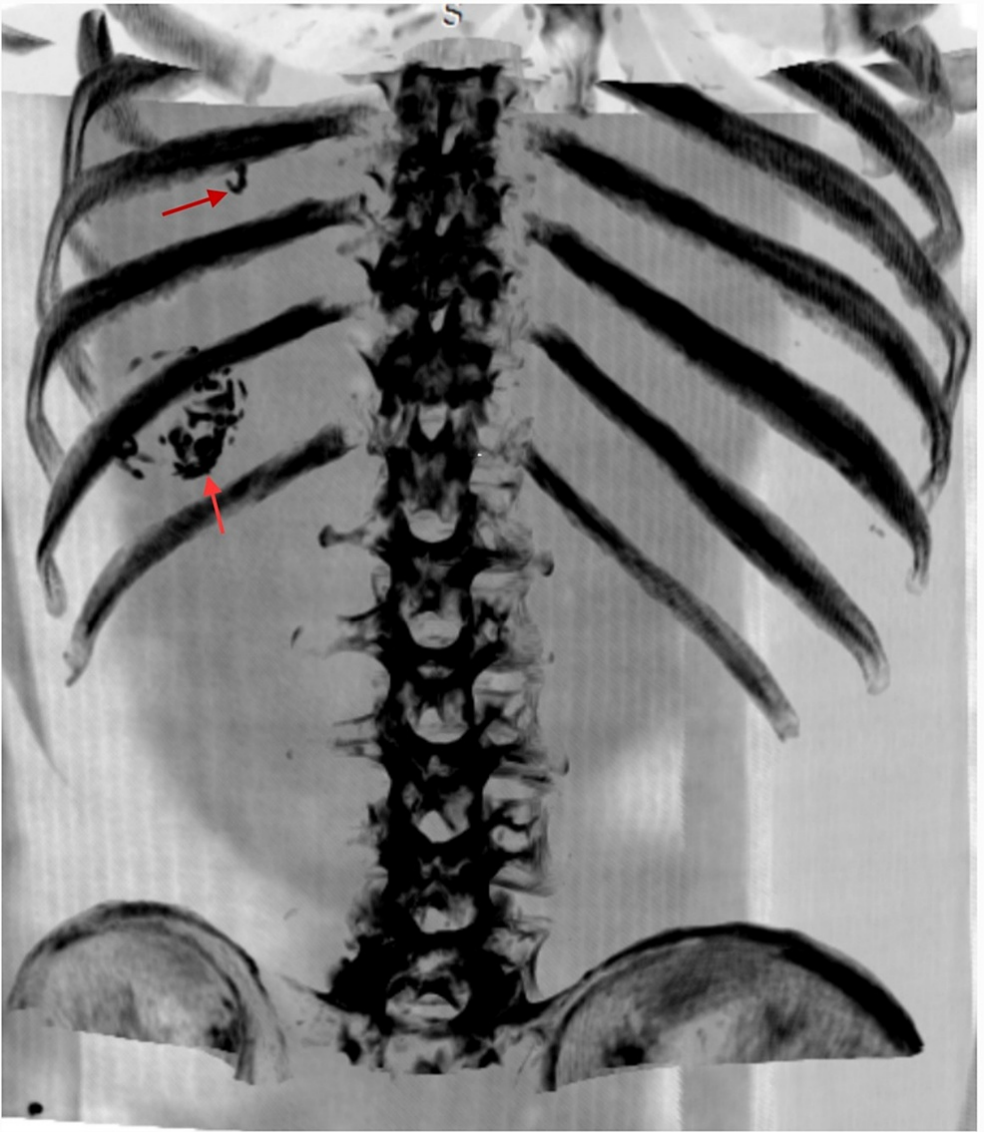
Maximum intensity projection (MIP) image showing inactive cyst. MIP image showing inactive cyst with circumferential calcification at the level of 11th rib. Another incomplete ring of calcification is seen at the level of ninth rib (which is a part of primary cyst and indicates active hydatid cyst). An important point, which is to be noted here, is that due to transdiaphragmatic spread of the hepatic hydatid into the adjacent lung it can mimic pulmonary hydatid on chest x-ray but suspicion can raise when the pulmonary hydatid is calcified, which is extremely rare. So, for any case of calcified pulmonary hydatid cyst further evaluation is to be warranted to look for transdiaphragmatic spread of hepatic hydatid.

**Figure 5:**
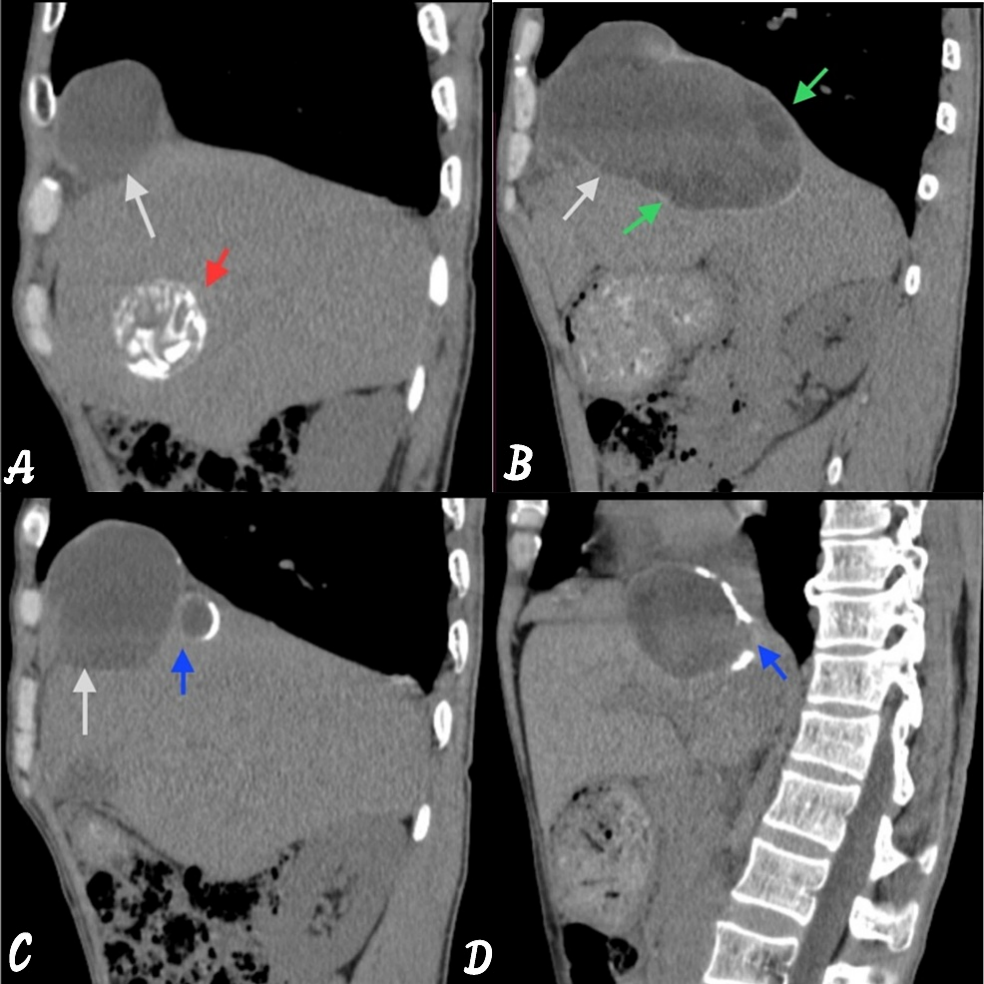
Sagittal reformatted images showing anterior transdiaphragmatic spread of the hydatid cyst. The image is showing (A) transdiaphragmatic spread of the hydatid cyst via segment VII, VIII, and IVA (white arrow) with dead calcified cyst (red arrow); (B) multiple daughter cysts within it (green arrow); (C and D) small cystic area with incomplete discontinuous calcification.

**Figure 6:**
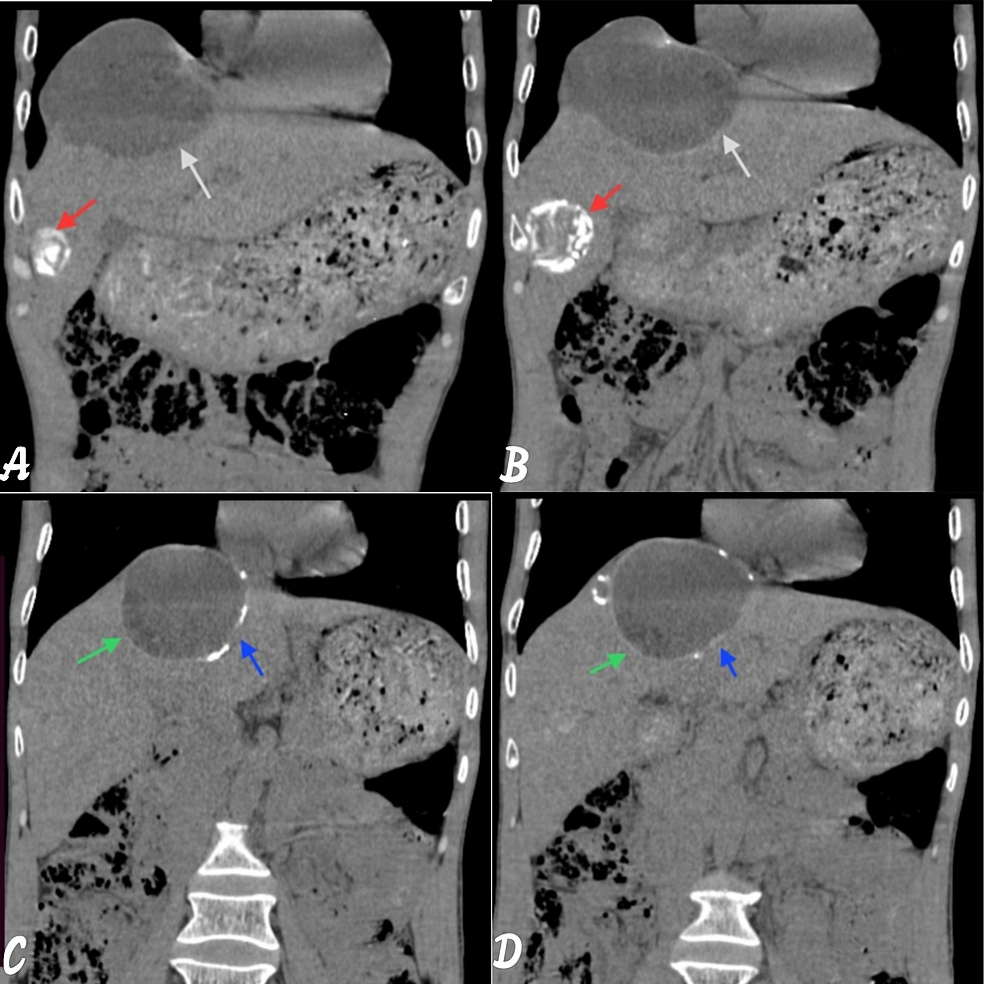
Coronal reformatted images showing both the active and the inactive cyst. (A and B) The active cyst is having transdiaphragmatic spread via segment VIII and IVA, another cyst is seen in segment V of liver and showing continuous whorls of calcifications likely inactive/dead cyst. (C and D) The active cyst has discontinuous incomplete calcifications (blue arrow) with multiple daughter cysts (green arrow) in the periphery.

To see for any extension and impending rupture, NCCT chest and abdomen was done with axial, sagittal, and coronal reformatted images. On the NCCT scan, the lesion is reaching up to right atrium (Figures 3A-3D) above the level of ninth rib (Figure 4). It has become an area of concern for impending rupture into the lung (Figures 5A-5D) or into the pericardium (Figures 6A-6D).

**Figure 7:**
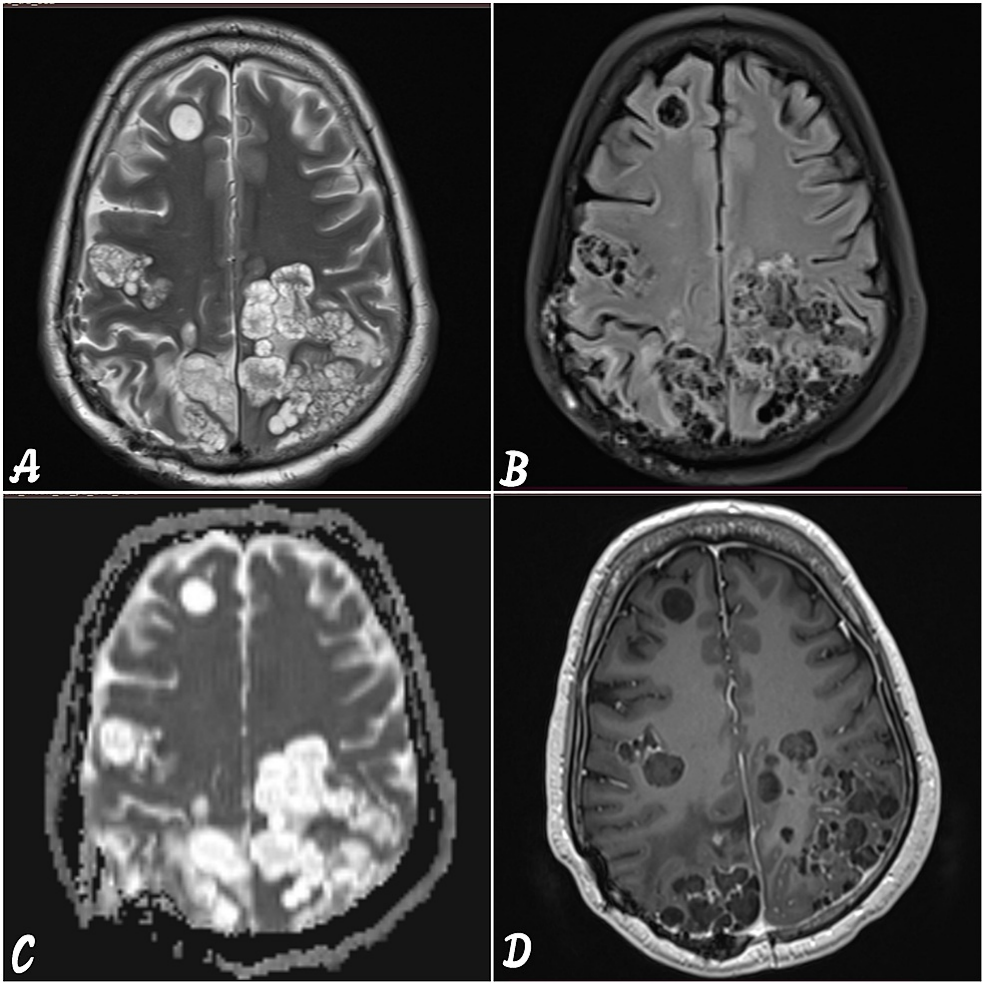
MRI brain with contrast (selected axial images). (A) T2-weighted image showing persistent cluster of thin-walled cystic lesions appearing hyperintense. (B) Fluid-attenuated inversion recovery (FLAIR) is showing hypointense signal in bilateral cerebral hemisphere (frontoparietal region and left occipital region). (C) Diffusion-weighted images (DWI) show no diffusion restriction. (D) T1 post-contrast image shows mild peripheral perilesional enhancement in bilateral cerebral hemispheres.

To look for the cause of focal neurological deficit and headache, magnetic resonance imaging (MRI) brain is done and it shows a cluster of discrete and confluent multiseptated cystic lesions appearing hyperintense on T2-weighted images (T2WI) and hypointense on T1-weighted images (T1WI) in the bilateral cerebral hemisphere in frontoparietal region and left occipital region (Figures 7A-7D). They show smooth peripheral enhancement on post-gadolinium images (Figures 7A-7D).

## Discussion

Hepatic disease

The right lobe is the most frequently involved portion of the liver. Imaging findings in hepatic hydatid disease depend on the stage of cyst growth [6]. The various staging system of hydatid cyst described in the literature are as follows: (1) based on morphology, the cyst can be classified into four different types - type I, simple cyst with no internal architecture; type II, cyst with daughter cyst(s) and matrix (IIa: round daughter cysts at the periphery, IIb: larger, irregularly shaped daughter cysts occupying the entire volume, IIc: oval masses with scattered calcifications and occasional daughter cysts); type III, calcified cyst (dead cyst); type IV, complicated cyst, e.g., a ruptured cyst. (2) The Gharbi ultrasound classification consists of five stages - stage 1, homogeneously hypoechogenic cystic thin-walled lesion; stage 2, septated cystic lesion; stage 3, cystic lesion with daughter lesions; stage 4, pseudo-tumor lesion; stage 5, calcified or partially calcified lesion (inactive cyst).

The above two classification systems are widely accepted and used but there can be suggestions and corrections that can be made to the classification system, e.g., stage 2 of Gharbi classification defines septated cystic lesion which can be modified to septation/localized split in the wall of cyst or partial floating membranes. All of these can be seen as echogenic septation within the cyst. Endo-cyst can become free-floating due to detachment from the peri-cyst which can be due to response to therapy, trauma, due to leakage or rupture of the hydatid cyst [6]. Complete detachment or rupture of the membranes within the cyst has been referred to as the water-lily sign and should not be included in modified stage 2 as it represents intracystic rupture [6].

Stage 3 of Gharbi classification and stage IIa of morphological classification can give a spokes wheel or cyst within the cyst appearance. The matrix has mixed echogenicity due to scolices, and hydatid sand [6]. So, the classifications can be modified by using the terms cyst within cyst or spoke wheel appearance with a mixed echogenicity hydatid sand. Stage 4 of Gharbi classification (the pseudotumor lesion) can have an appearance similar to a hepatic abscess or hepatization and is difficult to differentiate.

Stage 5 of Gharbi classification defines calcified or partially calcified cyst into one which is incorrect as only completely calcified curvilinear walls of calcification signify the death of the parasite [6]. Another setback is when the cyst is heavily calcified and only the anterior portion is visualized with posterior acoustic shadowing which can be misdiagnosed into a completely calcified cyst [6].

## Conclusions

This case highlights a rare presentation in which hepatic hydatid disease has not only led to transdiaphragmatic spread via segment VII of the liver into the adjacent lung but also distal hydatidosis into the cerebral parenchyma. The valid explanation for cerebral involvement is via the hematogenous spread of hydatid disease which can occur in any anatomic location (except hair, nail, etc.).

An interesting point that this research article has highlighted is that hepatic hydatid disease due to transdiaphragmatic spread can reach as high as eighth to ninth rib in the lung and can be confused as pulmonary hydatid cyst as in our case. But one can get suspicious if the pulmonary hydatid cyst is calcified as calcification of Pulmonary hydatid is extremely rare. Chest x-ray findings can be confusing as pulmonary calcified hydatid cysts are extremely rare. So, The unusual presentation of calcification within the lung hydatid cyst should always be further evaluated keeping in mind that it could be a complication of hepatic hydatid cyst by its transdiaphragmatic spread.

Another point that I want to highlight is that early diagnosis in these cases is a must and should be warranted as hematogenous spread can lead to poor prognosis and worse outcomes. Intradermal skin tests and thorough clinical history should be taken to make an early diagnosis and prevent the dissemination of the hydatid disease. Dissemination into the vital organs like the brain and heart can prove fatal and further evaluation should be done in suspicious/rare cases, for example, calcified pulmonary hydatid cyst. The area of weakness at both radiology and clinical level that I want to highlight in this article is that how a common disease can turn into a fatal one. In the end, atypical manifestations of hydatid disease though rare should always be considered for making an accurate and early diagnosis.
